# Insulin Resistance is Associated with Higher Plasma Viral Load Among HIV-Positive Adults Receiving Longer-Term (1 Year) Combination Antiretroviral Therapy (ART)

**Published:** 2019-08-16

**Authors:** LB Mulenga, P Musonda, L Chirwa, M Siwingwa, A Mweemba, S Suwilanji, S Fwoloshi, H Phiri, D Phiri, PL Mulenga, T Chisenga, R Nsakanya, A Shibemba, J Todd, S Nzala, T Kaile, C Kankasa, L Hachaambwa, C Claassen, I Sikazwe, JR Koethe, E Sinkala, DC Heimburger, CW Wester

**Affiliations:** 1 University of Zambia, School of Medicine, Division of Infectious Diseases, Internal Medicine, Lusaka, Zambia; 2 University Teaching Hospital, Adult Infectious Diseases Center, Lusaka, Zambia; 3 Ministry of Health, Ndeke House, Lusaka, Zambia; 4 Vanderbilt University Medical Center (VUMC), Department of Medicine, Division of Infectious Diseases, Nashville, TN, USA; 5 London School of Hygiene and Tropical Medicine, London, United Kingdom; 6 University of Maryland, Baltimore, MD, USA; 7 University of Alabama at Birmingham, Birmingham, AL, USA; 8 Centre for Infectious Diseases Research, Lusaka, Zambia; 9 Vanderbilt Institute for Global Health (VIGH), Nashville, TN, USA

**Keywords:** Insulin resistance, Combination antiretroviral therapy, Viral suppression, Treatment failure, Zambia

## Abstract

**Background:**

As HIV-positive persons survive longer due to the success of combination antiretroviral therapy (ART) in decreasing mortality, the burden of non-communicable diseases including diabetes mellitus (DM) is anticipated to rise. HIV is characterized by systemic inflammations, markers of which decrease quickly following ART initiation, but typically do not completely normalize. Inflammation may be accompanied by insulin resistance (IR), and both are implicated in the pathogenesis of DM in HIV-positive individuals. Sub-Saharan Africa accounts for almost two-thirds of the global HIV burden but there are few reports of IR, DM and HIV in this region. We assessed the relationship between IR and viral suppression among HIV-positive adults in the Zambian national ART program.

**Methods:**

We conducted a cross-sectional survey evaluating HIV-positive adults that had received first line ART (usually TDF/FTC/EFV) for 12 months (± 3 months). Twenty clinics were sampled systematically based on the random starting-point, sampling interval and cumulative population size. Eligible patients had plasma viral load (VL), fasting insulin, and glucose performed. Insulin resistance was determined using Homeostatic model assessment (HOMA). We determined proportions for each outcome using linearized standard error 95% confidence intervals and summary estimates. Viral suppression was defined according to the detection threshold of<20 copies/mL and treatment failure was defined as VL>1,000 copies/mL.

**Results:**

Of 473 patients enrolled, 46.8% were male and 53.2% were female. 142 (30%) [95% CI: 0.26–0.34] had IR. Among those with IR, 55 (38.7%) were male whereas 87 (61.3%) were female (p value=0.104). 19% of individuals with IR had treatment failure compared to 5.7% without IR (p value<0.0001). 427 (90.3%) participants had treatment success (VL<1,000 copies/mL), and this was associated with a lower likelihood of IR (odds ratio (OR)=0.26 [0.14, 0.48], p value<0.0001). In addition, a significantly lower proportion of patients with IR were virologically suppressed at one-year compared to individuals without IR, 58% [0.54–0.70] versus 70% [0.65–0.75], respectively (p value=0.042).

**Conclusion:**

In Zambian adults on ART for a year, the development of insulin resistance was strongly associated with suboptimal HIV outcomes, specifically non-viral suppression and treatment failure. Further investigations are warranted to determine if this positive association between IR and VL is causally related, and if so in which direction.

## Introduction

HIV continues to be a major public health concern around the globe especially within sub-Saharan Africa where an estimated 25.6 million of the global 36.7 million people living with HIV reside [1]. Over the past 10+ years, there has been an unprecedented scale-up in the provision of combination antiretroviral therapy (ART) to persons living with HIV; including the disproportionately affected region of sub-Saharan Africa.

This unprecedented scale-up of ART has been a major contributing factor to the 48% decline in AIDS-related deaths witnessed between 2005 and 2016 (UNAIDS 2017). However, as life expectancy increases, HIV-positive individuals are increasingly experiencing co-morbid medical conditions; which are age-related [2,3]. These age-related co-morbid medical conditions are anticipated to rise dramatically over the next 20 years, particularly in Sub-Saharan Africa where communicable diseases are being surpassed by non-communicable diseases (NCDs) as causes of premature mortality [2–10]. Furthermore, these NCDs due to their intersection with HIV, if not controlled in resource-constrained settings of the world, would have not only individual health consequences but also significant deleterious economic implications [7,9].

Diabetes mellitus is one of the most prevalent NCDs globally and the number of individuals developing diabetes mellitus in Africa is anticipated to increase substantially by 2030 [11,12]. Data from the USA and Europe shows that HIV-positive, ART-treated men have a higher incidence of DM when compared to match HIV-negative counterparts [13–15]. A higher prevalence of DM (18%) has also been reported among HIV-positive, ART-naïve adults in Tanzania, compared to HIV-negative adults (5.2%) residing in the same locality.

HIV infection itself may be associated with DM as HIV-positive individuals can develop metabolic syndrome, altered glucose metabolism, dyslipidaemias and lipodystrophy[16]. Furthermore, exposure to ART including; protease inhibitors (PIs) [e.g. indinavir (IDV), lopinavir (LPV)], and some nucleoside reverse transcriptase inhibitors (NRTIs) [e.g. zidovudine (AZT), stavudine (d4T), didanosine (ddI)] may also be contributing factors[17]. Other studies have implicated ART via autoimmune mediated immune reconstitution as an indirect cause of DM [18–20].

Insulin resistance (IR), rather than insulin deficiency is thought to be the main driver of DM among HIV-positive adults, a mechanism also observed in persistent Hepatitis C virus (HCV) infection associated DM [21,22]. In HIV-positive adults, IR may occur independently of ART exposure, but is likely due to persistent immune activation/inflammation [21–24].

Understanding the burden of IR among HIV-positive individuals, and its associated immunologic and virologic factors is critically important within sub-Saharan Africa, where the vast majority of HIV-positive individuals on ART reside. Such an understanding of these adverse metabolic effects including insulin resistance, dyslipidemia, and inflammation would provide invaluable information informing the design of effective interventions to prevent the development of diabetes mellitus in this aging “at risk” population. In this study, we assessed the relationship between IR and viral suppression among HIV-positive adults on ART in the Zambian HIV program.

## Materials and Methods

### Methodology

We conducted a cross-sectional study evaluating HIV-positive adults that had received first line ART for twelve (12) months with a margin of ± 3 months added to the time point to improve feasibility. A two-stage cluster design was employed with i) 20 clinics being randomly sampled from all clinics dispensing ART in the country and ii) patients being enrolled if they were on ART for 12 months.

In brief, sampling of clinics was performed using systematic sampling to generate probability proportional to proxy size samples. The probability proportional to size (PPS) sampling was used in order to have patients in large sites have the same probability of being selected as patients in smaller sites, and vice versa.

Eligible patients on ART for 12 months were recruited from each of the selected clinics and had fasting blood specimens obtained for CD4 cell count, plasma HIV-1 RNA (viral load), high sensitivity C-Reactive Protein (hsCRP), Tumor Necrosis Factor-Alpha (TNF-α), insulin, glucose, electrolytes including kidney function (chemistry), serum lipids and Complete Blood Count (CBC) testing. Specimens having plasma VL levels ≥ 1,000 copies/mL were genotyped to determine HIV drug resistance (HIVDR) status.

The collected blood was delivered to the University Teaching Hospital (UTH) laboratory for VL, hsCRP, TNF-α, Insulin, chemistry and serum lipid testing. CD4 cell count, glucose and complete blood counts (CBC) were performed at the local testing labs. No dried blood spots were collected due to lack of expertise to analyze these samples.

Viral loads were measured by polymerase chain reaction (PCR) using the COBAS® AmpliPrep/TaqMan® HIV-1 Qualitative Test, version 2.0 (TaqMan 96) assay. Virologic definitions included the following: i) viral suppression [10] was defined as having a VL<20 copies/mL; ii)non-viral suppression (NVS) defined as having a VL value ≥ 20 copies/mL; iii) treatment failure (TF) as having a VL value ≥ 1,000 copies/mL; and iv) treatment success (TS) being defined as having a VL<1,000 copies/mL.

Insulin was measured using the human insulin (Hu Insulin™) ELISA Kit and IR was measured using the Homeostasis Model Assessment of IR (HOMA-IR) test, a test widely accepted as a surrogate of IR in numerous populations[25,26]. IR was defined as having a HOMA ≥ 2 as reported best HOMA-IR cut-off levels range from 1.85 to 2.07. The Biosource Sensitivity Immunoassay (EASIA)™ for TNF-α was used and performed on a microtiter plate [27].

All analyses were performed using Stata Statistical Software: Release 15. College Station, TX (StataCorp LLC) to determine proportions for each outcome including linearized standard error 95% confidence intervals and summary estimates. Associations of insulin resistance, inflammatory markers, and treatment failure were measured using the chi-square or fishers exact test. A multivariable logistic regression analysis was used to predict factors associated with insulin resistance.

## Results

Four hundred and seventy-three (473) individuals were recruited in the study from all twenty facilities of which 46.8% were male and 53.2% were female. The study design-weighted mean time on ART was 12.2 months (95% CI: 11.8–12.6).

Thirty percent (30%), (95% CI: 26%−34%) of individuals had IR and there was no age difference between patients with and without IR. Patients with IR had increased waist circumference, waist-to-hip circumference ratio and increased visceral fat (Table 1). Other characteristics that varied between the two groups were fasting glucose, viral load, BMI, HDL cholesterol, LDL cholesterol, triglycerides, hsCRP and TNF-α levels/values (Table 2).

Sex was not associated with IR, 55 (38.7%) males had IR versus 87 (61.3%) females; p value=0.104. A significantly lower proportion of patients with IR were virologically suppressed at one-year compared to individuals without IR, 82 (58%) of 142 patients [95% CI: 0.54–0.70] versus 232 (70%) of 331 patients [95% CI: 0.65–0.75], respectively; p value=0.042.

When analyzed by VL threshold, specifically treatment success versus treatment failure, a significantly higher (3.3-fold) proportion of individuals with IR (19%) had treatment failure compared to patients without IR (5.7%); p value<0.0001. Of note, 427 (90.3%) participants had treatment success, and this was associated with a significantly lower (74% lower risk) likelihood of developing IR (odds ratio (OR)=0.26 [0.14, 0.48], p value<0.0001.

Among those without evidence of viral suppression, there was an increased odds of having IR; specifically, OR=1.44 (0.95, 2.17), p value 0.048). Similarly, the waist-to-hip circumference ratio was also associated with increased odds of IR.

A number of models were run including

1. Age, Waist circumference, Visceral fat, CD4, Hgb, Viral Load (VL), Triglycerides/HDL ratio, CRP, TNF alpha, Sex, Non-Viral Suppression (NVS) (VL ≥ 20 c/mL)
2. Age, Waist to Hip circumference ratio, Visceral fat, CD4, Hgb, VL, Triglycerides/HDL ratio, CRP, TNF alpha, NVS
3. Age, BMI, Visceral fat, CD4, Hb, VL, LDL, CRP, TNF alpha, NVS and
4. Age, BMI, Visceral fat, CD4, Hb, VL, Triglycerides/HDL ratio, CRP, TNF alpha, NVS

Using the best adjusted model (Table 3), NVS, TNF-α, CRP, waist-to-hip ratio and visceral fat were associated with increased odds of IR.

## Discussion

In this HIV-positive population of Zambian adults who have been receiving combination antiretroviral therapy (ART) for a mean time of 12.2 months, 30% of them had insulin resistance (IR) as ascertained using the Homeostatic Model Assessment (HOMA). This is the first time IR has been studied in Zambia and also in the region among individuals who are HIV-positive and currently receiving ART. When analyzed by VL threshold, specifically, treatment success and treatment failure; 19% of individuals with IR had treatment failure compared to 5.7% without (p value<0.0001). The vast majority (90.3%) of study participants had treatment success and this was associated with a significantly lower (74% lower risk) likelihood of developing IR (OR=0.26 [0.14, 0.48], p value<0.0001.

Using the VL threshold of<20 copies/mL to define viral suppression (Murray, Vos et al.), this study found that a significantly lower proportion of patients with IR were virologically suppressed at one-year compared to individuals without IR, 58% [0.54–0.70] versus 70% [0.65–0.75], p value=0.042. Individuals with viremia or NVS had increased odds (OR 1.44, 95%CI: 0.95–2.17, p=0.048) and those with treatment success (showed a lower likelihood of IR; Odds ratio (OR)=0.26 [0.14, 0.48], p value<0.0001.

These findings are interesting in that they provide some insight into the possibility that the risk for the development of IR may be diminished in patients with both viral suppression and treatment success. Since individuals in this study were on ARV medications that have not been implicated in the development of IR, we could not explicitly explain these findings based on viral suppression and treatment success.

The percentage of individuals with IR is comparable to studies performed among different populations and regions of the world [24,28,29]. This study showed similar results to a study conducted in Spain where 265 HIV-positive adults on ART without DM at baseline had an IR prevalence of 21% [30]. A similar study in Cameroon, Central Africa showed a much higher prevalence of 47.3 among HIV-positive adults (48.5% among HIV-positive, ART-treated adults versus 38.5% among ART-naïve adults) compared to our study. This particular study was hospital-based and even though the majority (93.3%) of patients on ART were receiving 1st line regimens, their ARV medications were different and the total duration on ART of their study population was also longer (i.e. 72 months) [31]. These discrepancies in the duration of treatment and ART regimens would also explain the difference in results, as exposure to certain ARV medications has also been implicated in the development of IR [18,19,22]. The ARV medications this Cameroonian population was primarily exposed to; namely protease inhibitors and thymidine analogues are the ARV medications which have largely been implicated in IR and DM [22,32].

The trend of increased inflammatory markers (highly sensitive C-reactive protein (hsCRP) and tumor necrosis factor-alpha (TNF−α) for those patients with IR may imply that inflammation may be associated with the development of IR in patients with HIV. In our study, the median hsCRP levels were 2.38 mg/L (95%CI: 1.40, 3.49) for those with IR compared to 0.79 mg/L (95%CI: 0.47, 1.58) for those without IR; p value<0.0001. Similarly, TNF-α levels were 33.9 pg/mL (95%CI: 27.5, 42.2) *Vs* 21.7 pg/mL (95%CI: 14.9, 29.9) among those study participants with and without IR, respectively. Using quantile regression, the risk for the development of IR appeared to be positively correlated with increasing hsCRP and TNF-α levels when evaluating these biomarker values as continuous covariates. Using logistic regression coefficients estimates of log odds of IR association with hsCRP and TNF-α, CRP was significantly associated with IR (0.431; p value<0.0001).

Though our study does not prove whether or not inflammation is the cause of IR, it does highlight the importance of the interdependence between inflammation and IR. As reported by Banks et al when evaluating the relationship between TNF-α and hsCRP with risk for IR, our study showed a similar positive association [33,34]. Other authors have suggested that in HIV, an infectious stimuli may result in persistent cytokine activation leading to prolonged release of TNF- and other inflammatory cytokines that lead to and affect the risk for the development of IR [24]. This phenomenon may be present even in the absence of exposure to ARV medications typically associated with IR risk [24]. It is therefore possible that our findings may be explained by an active infectious stimuli (uncontrolled HIV) in viremic individuals, both lower levels (VL>20 copies/mL) and higher levels (VL>1,000 copies/mL) of viremia leading to varying levels of cytokine activation with the extensive and prolonged release of the TNF- and other inflammatory cytokines with subsequent reduction in insulin sensitivity. Insulin resistance may also be due to the HIV-1 accessory proteins of Tat and Vpr with Vpr obstructing the transcriptional activity of insulin through inhibition of the PPAR-c activity and Tat activating the nuclear factor−κβ with induction of TNF- and blocking of the uptake of free fatty acids by adipocytes [24,34,35]. The fact that markers of inflammation are decreased on ART may explain the observed lower levels in patients with viral suppression and also reduced odds for risk of developing IR [36–38]. It is thus plausible that controlling HIV infection with ART may be protective towards IR and this may be a result of reduced inflammation in virally suppressed individuals.

Besides inflammatory markers and NVS, we observed that individuals with increasing waist-to-hip circumference ratio, body mass indices, visceral fat levels, and levels of LDL cholesterol and triglycerides had an increased odd of developing IR. We observed increased odds of developing IR among individuals with increased visceral fat levels and increased Waist-to-Hip circumference ratios suggesting that visceral fat in HIV-positive individuals may be a predictor of IR. Hadigan et al described IR in HIV-positive adults and demonstrated significant hyperinsulinemia and truncal adiposity in HIV-positive women independent of PI exposure [38–40]. IR and dyslipidemia are common metabolic disorders among PLWH either directly (i.e. for dyslipidemia) and indirectly (i.e., HIV-associated inflammation) and/or ART (i.e. especially older generation ART regimens) [41]. There are still ongoing studies evaluating the specific link between visceral adipose tissue accumulation and IR. Suggested mechanisms are largely centered upon insulin signaling and may be related to: a) the accumulation of excess lipid in liver resulting in cell autonomous impairment in insulin signaling and b) visceral adipose tissue inflammation and inflammatory cytokine production contributing to impairment in insulin signaling [42–44].

These data demonstrate significant rates of IR in HIV-positive, ART-treated adults having both increased visceral fat levels and waist-to-hip circumference ratios, and based on this a simpler and certainly less invasive means to monitor for IR; may be to regularly perform WHRs among at-risk individuals. Performing WHRs at some frequency may also be a way to diagnose additional adverse health events such as diabetes mellitus and cardiovascular disease. This monitoring may be especially critical in HIV-positive adults where a variety of mechanisms such inflammation, ART regimen exposure, and HIV are simultaneously interacting and have been linked with the development of IR, DM and cardiovascular disease.

We believe this is the first study to highlight that early ART leading to virologic suppression may reduce one’s risk for the development of IR. Our study is in contrast to the Rwanda study which was done among HIV-positive women where ART use did not predict change in log10-HOMA and use or duration of AZT, d4T and EFV was not associated with HOMA change [45]. This could be due to differences in ARV medications used, and was primarily done in a female population, and the investigative team did not use viral load to determine treatment response.

Both bacterial and viral infections have been associated with reduced insulin sensitivity and similar to our findings, risk for development of IR may be independent of exposure to certain ARV medications or classes of medications (e.g. PIs) [24,46]. Assessing the prevalence of IR in a Spanish cohort of HIV-positive adults, Araujo et al. attributed the lower prevalence of IR to participants in their cohort receiving newer antiretroviral medications [30]. It is therefore plausible to have different IR levels and risk depending on the type of ARV medications used. Previously used ARV medications (i.e. d4T, AZT, ddI, etc.) were associated with IR and persons receiving them also had more medication-related side effects such as lipodystrophy, both of which have been implicated in IR emergence. Additionally, HIV-positive adults receiving these older ARV regimens may be more likely to be non-adherent due to unfavorable side effects and/or significant pill burden that would lead to suboptimal virologic suppression that could directly influence insulin sensitivity. Our study, however, suggested that ART itself did not have any direct influence on the development of IR in contrast to what has been reported elsewhere [30].

Since ARV medications themselves may be linked to IR, it is therefore critical that the selection of ART regimens is taken into account when deciding upon what ART regimens to initiate (or switch to) at the individual patient-level. Recent reports of the newly introduced integrase strand inhibitor dolutegravir (DTG), that is being rolled out to majority of individuals on ART being linked to increasing weight, are concerning and warrant further study. Recently, Venter and group reported increased weight on DTG based therapy in south African HIV positive adults much more in female [47,48]. These recent data that Menard et al describe as “an unexpected bothering side effect” are worrisome as DM rates may increase independently of the metabolic syndrome and as a result there may be more intermediate-and longer-term risks associated with DTG exposure than previously elucidated [48–50]. Our study participants were not on DTG-based ART, thus we could not study these potential causal relationships and certainly additional studies are needed.

Our study has several limitations. 1) This was a cross sectional study and thus we could not establish pre-treatment/baseline levels of IR in our population and other pre-treatment parameters such as viral load, HIVDR, visceral fat levels and anthropometric indices. 2) Our team did not have an HIV-negative control group or an HIV-positive, ART-naive group which would have helped to disentangle the role of HIV and ART exposure in the development of and progression of IR.

## Conclusion

In summary, we have shown that insulin resistance rates in HIV-positive adults receiving ART for 1 year are high and associated with both non-viral suppression (viral load>20 copies/mL) and treatment failure (viral load>1,000 copies/mL). Insulin resistance was further associated with high inflammatory markers (hsCRP and TNF-α), visceral fat and obesity. These findings may have wider implications for a number of reasons:

Individuals with HIV are now living longer and are at high risk for NCDs including diabetes mellitus.

People Living with HIV (PLHIV) have systemic inflammation (as evidenced in this study), another factor associated with type 2 diabetes.

PLHIV may have lip dystrophic features with increasing obesity related to both HIV infection and ART exposure, both of which are related to incident type2 diabetes.

The wide scale introduction of DTG, with improved potency (i.e. higher viral suppression rates) and tolerability, may be associated with obesity which has been linked to IR and incident DM.

## Figures and Tables

**Table 1:** Characteristics of individuals with or without insulin resistance

	Insulin Resistance		
Characteristics	No N=331 (70%)	Yes N=142 (30%)	p value
Continuous variables (Median, IQR)			
Age (years)	37 (32,44)	36 (29,44)	0.064W
Waist circumference (cm)	81 (74,88)	87 (78.5,94)	<0.0001W
Hip circumference (cm)	94 (88,100)	90 (79,97)	0.0002W
Waist to Hip circumference ratio	0.85 (0.8,0.92)	0.99 (0.88,1.10)	<0.0001W
Visceral Fat Level	5 (3,8)	11 (4,14)	<0.0001W
CD4+ cell count (cells/μL)	346 (221,547)	391 (264,593)	0.097W
Hemoglobin (g/dl)	13.0 (11.8,14.2)	12.8 (11.5, 14.3)	0.349W
Fasting Blood Glucose (mmol/l)	5.40 (5.00, 6.00)	5.90 (5.40, 6.50)	<0.0001W
Viral load (copies/mL)	20 (20,38)	20 (20,176)	0.011W
Body Mass Index (kg/m2)	21.8 (19.7,24.7)	22.7 (20.2,25.6)	0.046W
Total Cholesterol (mmol/l)	4.2 (3.49,4.80)	4.42 (3.64,5.19)	0.085W
High Density Lipoprotein (mmol/l)	1.99 (1.32,2.51)	1.00 (0.74,1.43)	<0.0001W
Low Density Lipoprotein (mmol/l)	1.90 (1.30,2.60)	2.24 (1.71,2,92)	0.0003W
Triglycerides (mmol/l)	0.95 (0.58,1.67)	2.16 (0.94,2.99)	<0.0001W
C-reactive protein (hsCRP) (mg/L)	0.79 (0.47,1.58)	2.38 (1.40,3.49)	<0.0001W
Tumor necrosis factor alpha (TNF-α)(pg/mL)	21.7 (14.9, 29.9)	33.9 (27.5,42.2)	<0.0001W
Insulin (mIU/L)	4.64 (2.81, 6.50)	34.5 (22.8,32.2)	<0.0001W
Categorical variables			
Type of facility Health center Hospital	196 (59.2) 135 (40.8)	85 (59.9) 57 (40.1)	0.490C
Sex Male Female	155 (46.8) 176 (53.4)	55 (38.7) 87 (61.3)	0.104C
Marital status (N=462) Single Cohabiting Married Divorced Widowed	43 (13.2) 7 (2.2) 204 (62.8) 25 (7.7) 46 (14.2)	20 (14.6) 2 (1.5) 77 (56.2) 24 (17.5) 14 (10.2)	0.033E
Employment status Unemployed Employed	189 (57.1) 142 (42.9)	90 (63.4) 52 (36.6)	0.203C
Treatment Supporter available No Yes	50 (15.1) 281 (84.9)	10 (7.0) 132 (93.0)	0.016C
WHO clinical stage 1 2 3 4	294 (92.5) 17 (5.4) 6 (1.9) 1 (0.3)	128 (94.1) 6 (4.4) 1 (0.7) 1 (0.7)	0.708E
ART Regimen ABC/3TC/EFV TDF/XTC/ATV TDF/XTC/EFV TDF/XTC/NVP	5 (1.5) 1 (0.3) 323 (97.6) 2 (0.6)	1 (0.7) 0 (Stein Schalkwijk) 140 (98.6) 1 (0.7)	0.899C
Hepatitis B surface antigen Negative Positive	317 (95.8) 14 (4.2)	138 (97.2) 4 (2.8)	0.462C
Rapid Plasma Reagin (Syphilis) Negative Positive	301 (90.9) 30 (9.1)	126 (88.7) 16 (11.3)	0.458C
Non-Viral Suppression (NVS) (≥20 copies/mL HIV RNA) No Yes	232 (70.1) 99 (29.9)	82 (58.0) 54 (42.0)	0.042C

W: Wilcoxon/Mann-Whitney Test Rank-Sum Test; C: Chi Squared Test; E: Fisher’s Exact Test

**Table 2:** Covariates and their association/risk for development of insulin resistance

Covariates	Unadjusted OR (95% CI)	p value	Adjusted OR (95% CI)	p value
Continuous variables				
Age (years)	0.98 (0.96, 1.00)	0.053	0.67 (0.86, 1.12)	0.262
Waist circumference (cm)	1.05 (1.03, 1.07)	<0.0001	1.11 (0.72, 1.70)	0.639
Hip circumference (cm)	0.97 (0.95, 0.98)	<0.0001	0.81 (0.50, 1.32)	0.401
Waist-to-Hip circumference ratio	246.4 (54.7,1108.9)	<0.0001	NB	NB
Visceral Fat Levels (IU)	1.14 (1.09, 1.19)	<0.0001	0.88 (0.59, 1.31)	0.536
CD4 cell count (cells/μL)	1.00 (0.99, 1.00)	0.096	1.00 (0.99, 1.00)	0.312
Haemoglobin (g/dl)	0.94 (0.83, 1.05)	0.273	1.39 (0.68, 2.82)	0.365
Fasting Blood Glucose (mmol/l)	1.32 (1.11, 1.59)	0.002	2.76 (1.15, 6.59)	0.022
Viral load (copies/mL)	1.00 (0.99, 1.00)	0.074	1.00 (0.99, 1.00)	0.600
Body Mass Index (kg/ m 2)	1.04 (0.99, 1.09)	0.087	1.02 (0.54, 1.93)	0.945
Total Cholesterol (mmol/l)	1.01 (0.94, 1.09)	0.729	0.96 (0.32, 2.87)	0.940
High Density Lipoprotein (mmol/l)	0.33 (0.25, 0.44)	<0.0001	2.42 (0.40, 14.7)	0.336
Low Density Lipoprotein (mmol/l)	1.29 (1.07, 1.56)	0.008	1.92 (0.26, 14.1)	0.520
Triglycerides (mmol/l)	1.15 (1.06, 1.25)	0.002	1.17 (0.74, 1.86)	0.493
C-reactive protein (hsCRP)(mg/L)	1.55 (1.36, 1.77)	<0.0001	0.95 (0.49, 1.84)	0.875
Tumor necrosis factor alpha (TNF-α)(ng/mL )	1.00 (0.99, 1.00)	0.193	1.03 (0.91, 1.17)	0.635
Type of facility Health center Hospital	ref 0.97 (0.65, 1.45)	0.896	ref 0.41(0.01, 12.9)	0.611
WHO clinical stage 1 2 3 4	ref 0.81 (0.31, 2.10) 0.38 (0.05, 3.21) 2.30 (0.14, 37.0)	0.666 0.376 0.558	ref NB NB NB	NB NB NB
ART Regimen Others TDF/XTC/EFV	ref 1.73 (0.36, 0.8.27)	0.490	ref NB	NB
Hepatitis B surface antigen Negative Positive	ref 0.66 (0.21, 2.03)	0.465	ref NB	NB
Rapid Plasma Reagin (Syphilis) Negative Positive	ref 1.27 (0.67, 2.42)	0.459	ref NB	NB
Non-Viral Suppression (NVS) ( VL ≥20 copies/mL) No Yes Treatment Success (VL<1,000 copies/mL) No Yes	ref 1.44 (0.95, 2.17) ref 0.26 (0.14, 0.48)	0.048 <0.0001		

W: Wilcoxon/Mann-Whitney Test Rank-Sum Test; C: Chi Squared Test; E: Fisher’s Exact Test; NB: Values not estimated due to perfect prediction

**Table 3:** Best adjusted model for odds of insulin resistance

Odds Ratio of Insulin Resistance				
Characteristics	Unadjusted OR (95% CI)	p value	Adjusted OR (95% CI)	p value
Continuous variables				
Age (years)	0.98 (0.96, 1.00)	0.019	0.94 (0.89, 0.99)	0.014
Waist circumference (cm)	1.05 (1.03, 1.07)	<0.0001	1.05 (1.00, 1.11)	0.073 (F/M)
Hip circumference (cm)	0.97 (0.95, 0.98)	<0.0001	0.96 (0.92, 1.01)	0.087
Waist to Hip circumference ratio	246.4 (54.7, 1108.9)	<0.0001		
Visceral Fat Levels (IU)	1.14 (1.09, 1.19)	<0.0001		
CD4 cell count (cells per μL)	1.00 (0.99, 1.00)	0.096		
Hemoglobin (g/dl)	0.94 (0.83, 1.05)	0.273	1.22 (0.93, 1.59)	0.149
Viral load (copies /mL)	1.00 (0.99, 1.00)	0.074		
Body Mass Index (m/kg2)	1.04 (0.99, 1.09)	0.087		
Total Cholesterol (mmol/l)	1.01 (0.94, 1.09)	0.729		
High Density Lipoprotein (mmol/l)	0.33 (0.25, 0.44)	<0.0001		
Low Density Lipoprotein (mmol/l)	1.29 (1.07, 1.56)	0.008	1.74 (1.02, 2.94)	0.041
Triglycerides (mmol/l)	1.15 (1.06, 1.25)	0.002	1.21 (1.05, 1.40)	0.010
C-reactive protein (hsCRP)(mg/L)	1.55 (1.36, 1.77)	<0.0001		
Tumor necrosis factor alpha (TNF-α)(ng/mL)	1.00 (0.99, 1.00)	0.193	1.04 (1.01, 1.07)	0.022
Categorical variables				
Type of facility Health center Hospital	ref 0.97 (0.65, 1.45)	0.896		
Sex Male Female	ref 1.39 (0.93, 2.08)	0.105		
WHO clinical stage 1 2 3 4	ref 0.81 (0.31, 2.10) 0.38 (0.05, 3.21) 2.30 (0.14, 37.0)	0.666 0.376 0.558		
ART Regimen Others TDF/XTC/EFV	ref 1.73 (0.36, 0.8.27)	0.490		
Test for hepatitis B Negative Positive	ref 0.66 (0.21, 2.03)	0.465		
Test for syphilis Negative Positive	ref 1.27 (0.67, 2.42)	0.459		
Non-Viral Suppression (NVS) (VL ≥20 copies/mL) No Yes Treatment Success (VL<1,000 copies/mL) No Yes	ref 1.44 (0.95, 2.17) ref 0.26 (0.14, 0.48)	0.048 <0.0001		

W: Wilcoxon/Mann-Whitney Test rank-sum test; C: Chi Squared Test; E: Fisher’s Exact Test; NB: Values not estimated due to perfect prediction

## References

[1] Gupta RK, Jordan MR, Sultan BJ, Hill A, Davis DHJ (2012) Global trends in antiretroviral resistance in treatment-naive individuals with HIV after rollout of antiretroviral treatment in resource-limited settings: A global collaborative study and meta-regression analysis. The Lancet 380: 1250–1258. 10.1016/S0140-6736(12)61038-1 PMC3790969 22828485

[2] Justice AC (2010) HIV and aging: Time for a new paradigm. Curr HIV/ AIDS Rep 7: 69–76. 20425560 10.1007/s11904-010-0041-9

[3] Deeks SG (2011) HIV infection, inflammation, immunosenescence, and aging. Annu Rev Med 62: 141–155. 21090961 10.1146/annurev-med-042909-093756 PMC3759035

[4] Negin J, Cumming RG (2010). HIV infection in older adults in sub-Saharan Africa: Extrapolating prevalence from existing data. Bull World Health Organ 88: 847–853. 21076566 10.2471/BLT.10.076349 PMC2971515

[5] Joint United Nations Programme on HIV/AIDS. (2010) Global report: UNAIDS report on the global AIDS epidemic 2010. Unaids.

[6] Wallrauch C, Barnighausen T, Newell ML (2010) HIV prevalence and incidence in people 50 years and older in rural South Africa. S Afr Med J 100: 812–814 21414272 10.7196/samj.4181 PMC4726738

[7] Haregu TN, Setswe G, Elliott J, Oldenburg B (2014) National responses to HIV/AIDS and non-communicable diseases in developing countries: Analysis of strategic parallels and differences. J Public Health Res 3: 99. 25170505 10.4081/jphr.2014.99 PMC4140380

[8] Duffy M, Ojikutu B, Andrian S, Sohng E, Minior T, (2017) Noncommunicable diseases and HIV care and treatment: Models of integrated service delivery.Trop Med Int Health 22: 926–937. 28544500 10.1111/tmi.12901

[9] Murray CJL, Lopez Alan D (1996) The Global burden of disease: A comprehensive assessment of mortality and disability from diseases, injuries, and risk factors in 1990 and projected to 2020.World Health Organization.

[10] Murray CJ, Vos T, Lozano R, Naghavi M, Flaxman AD, (2012) Disability-adjusted life years (DALYs) for 291 diseases and injuries in 21 regions, 1990–2010: A systematic analysis for the Global Burden of Disease Study, 2010. Lancet 380: 2197–2223. 23245608 10.1016/S0140-6736(12)61689-4

[11] Whiting DR, Guariguata L, Weil C, Shaw J (2011) IDF diabetes atlas: Global estimates of the prevalence of diabetes for 2011 and 2030.Diabetes Res Clin Pract 94: 311–321. 22079683 10.1016/j.diabres.2011.10.029

[12] Faurholt-Jepsen D, Range N, Praygod G, Jeremiah K, Faurholt-Jepsen M, (2014) The association between conventional risk factors and diabetes is weak among urban Tanzanians. Diabetes Care 37: 5–6. 24356608 10.2337/dc13-1905

[13] Brown TT, Li X, Cole SR, Kingsley LA, Palella FJ, (2005) Cumulative exposure to nucleoside analogue reverse transcriptase inhibitors is associated with insulin resistance markers in the Multicenter AIDS Cohort Study. AIDS 19: 1375–1383. 16103768 10.1097/01.aids.0000181011.62385.91

[14] Brown TT, Cole SR, Li X, Kingsley LA, Palella FJ, (2005) Antiretroviral therapy and the prevalence and incidence of diabetes mellitus in the multicenter AIDS cohort study. Arch Intern Med 165: 1179–1184. 15911733 10.1001/archinte.165.10.1179

[15] Galli L, Salpietro S, Pellicciotta G, Galliani A, Piatti P, (2012) Risk of type 2 diabetes among HIV-infected and healthy subjects in Italy.Eur J Epidemiol 27: 657–665. 22722952 10.1007/s10654-012-9707-5

[16] Maganga E, Smart LR, Kalluvya S, Kataraihya JB, Saleh AM, (2015) Glucose metabolism disorders, HIV and Antiretroviral therapy among Tanzanian adults. PLoS One 10: e0134410. 10.1371/journal.pone.0134410 PMC4545793 26287742

[17] Kalra S, Kalra B, Agrawal N, Unnikrishnan A (2011) Understanding diabetes in patients with HIV/AIDS. Diabetol Metab Syndr 3: 2. 21232158 10.1186/1758-5996-3-2 PMC3025836

[18] Fleischman A, Johnsen S, Systrom DM, Hrovat M, Farrar CT, (2007) Effects of a nucleoside reverse transcriptase inhibitor, stavudine on glucose disposal and mitochondrial function in muscle of healthy adults. Am J Physiol Endocrinol Metab 292: 1666–1673. 10.1152/ajpendo.00550.2006 PMC3206591 17284576

[19] Blümer Regje ME, Marit VV, Jussi S, Elly H, Mariette A, (2008). Zidovudine: Lamivudine contributes to insulin resistance within 3 months of starting combination antiretroviral therapy. AIDS 22: 227–236. 18097225 10.1097/QAD.0b013e3282f33557

[20] Takarabe D, Rokukawa Y, Takahashi Y, Goto A, Takaichi M, (2010). Autoimmune diabetes in HIV-infected patients on highly active antiretroviral therapy.J Clin Endocrinol Metab 95: 4056–4060. 20484483 10.1210/jc.2010-0055

[21] Mehta SH, Brancati FL, Sulkowski MS, Strathdee SA, (2001). Prevalence of type 2 diabetes mellitus among persons with hepatitis C virus infection in the United States. Hepatology 33: 1554–1554. 11391549 10.1053/jhep.2001.0103306le01

[22] Dagogo-Jack S (2008) HIV therapy and diabetes risk. Diabetes Care 31: 1267–1268. 18509145 10.2337/dc08-0459

[23] Ji Kon R, Sang Bae L, Sa JH (2001) Association of chronic Hepatitis C virus infection and diabetes Mellitus in Korean patients. The Korean Journal of Internal Medicine. 10.3904/kjim.2001.16.1.18 PMC4531698 11417300

[24] Limone P (2003) Insulin resistance in HIV-infected patients: Relationship with pro-inflammatory cytokines released by peripheral leukocytes. Journal of Infection 47: 52–58. 12850163 10.1016/s0163-4453(03)00055-0

[25] C Lann D (2007) Insulin resistance as the underlying cause for the metabolic syndrome.Med Clin North Am 91: 1063–1077. 17964909 10.1016/j.mcna.2007.06.012

[26] Antuna-Puente B, Rabasa-Lhoret R, Laville M, Capeau J, Bastard JP, (2011) How can we measure insulin sensitivity/resistance? Diabetes Metab 37: 179–188. 21435930 10.1016/j.diabet.2011.01.002

[27] Gayoso-Diz P, Otero-Gonzalez A, Rodriguez-Alvarez MX, Gude F, Garcia F, (2013) Insulin resistance (HOMA-IR) cut-off values and the metabolic syndrome in a general adult population: Effect of gender and age: EPIRCE cross-sectional study. BMC Endocr Disord 13: 47. 24131857 10.1186/1472-6823-13-47 PMC4016563

[28] Guillen MA, Mejia FA, Villena J, Turin CG, Carcamo CP, (2015) Insulin resistance by homeostasis model assessment in HIV-infected patients on highly active antiretroviral therapy: Cross-sectional study. Diabetol Metab Syndr 7: 49. 26034512 10.1186/s13098-015-0046-z PMC4450995

[29] Viana CM, Ernesto R (2018) The prevalence of insulin resistance and related metabolic features in aruba-revelations, implications, and hope.

[30] Araujo S, Banon S, Machuca I, Moreno A, Perez-Elias MJ, (2014) Prevalence of insulin resistance and risk of diabetes mellitus in HIV-infected patients receiving current antiretroviral drugs. Eur J Endocrinol 171: 545–554. 25117462 10.1530/EJE-14-0337

[31] Noumegni SRN, Nansseu JR, Ama VJM, Bigna JJ, Assah FK (2017) Insulin resistance and associated factors among HIV-infected patients in sub-Saharan Africa: A cross sectional study from Cameroon. Lipids Health Dis 16: 148. 28797289 10.1186/s12944-017-0543-1 PMC5553591

[32] Vigouroux C, Maachi M, Nguyen TH, Coussieu C, Gharakhanian S, (2003) Serum adipocytokines are related to lipodystrophy and metabolic disorders in HIV-infected men under antiretroviral therapy. AIDS 17: 1503–1511. 12824788 10.1097/00002030-200307040-00011

[33] Banks WA, Willoughby LM, Thomas DR, Morley JE (2007) Insulin resistance syndrome in the elderly: Assessment of functional, biochemical, metabolic, and inflammatory status. Diabetes Care 30: 2369–2373. 17536070 10.2337/dc07-0649

[34] Kino T, Mirani M, Alesci S, Chrousos GP (2003) AIDS-related lipodystrophy/insulin resistance syndrome. Horm Metab Res 35: 129–136. 12734771 10.1055/s-2003-39072

[35] Leow MK, Addy CL, Mantzoros CS (2003) Clinical review 159: Human immunodeficiency virus/highly active antiretroviral therapy-associated metabolic syndrome: Clinical presentation, pathophysiology, and therapeutic strategies. J Clin Endocrinol Metab 88: 1961–1976. 12727939 10.1210/jc.2002-021704

[36] Brown TT, McComsey GA, King MS, Qaqish RB, Bernstein BM, (2009) Loss of bone mineral density after antiretroviral therapy initiation, independent of antiretroviral regimen. J Acquir Immune Defic Syndr 51: 554–561. 19512937 10.1097/QAI.0b013e3181adce44

[37] Brigham EP, Patil SP, Jacobson LP, Margolick JB, Godfrey R, (2014) Association between systemic inflammation and obstructive sleep apnea in men with or at risk for HIV infection. Antivir Ther 19: 725–733. 24518040 10.3851/IMP2745 PMC4130807

[38] Hadigan C, Miller K, Corcoran C, Anderson E, Basgoz N, .(1999) Fasting hyperinsulinemia and changes in regional body composition in human immunodeficiency virus-infected women. J Clin Endocrinol Metab 84: 1932–1937. 10372689 10.1210/jcem.84.6.5738

[39] Hadigan C, Stanley T, Piecuch S, Klibanski A, Grinspoon S (2000) Fasting hyperinsulinemia in human immuno-deficiency virus-infected men: Relationship to body composition, gonadal function, and protease inhibitor use. J Clin Endocrinol Metab 85: 35–41. 10634360 10.1210/jcem.85.1.6264

[40] Hadigan C (2005) Insulin Resistance among HIV-Infected Patients: Unraveling the Mechanism. CID 41: 1341–1342. 10.1086/496990 16206113

[41] Non LR, Escota GV, Powderly WG (2017). HIV and its relationship to insulin resistance and lipid abnormalities. Transl Res 183: 41–56. 28068521 10.1016/j.trsl.2016.12.007

[42] Hardy OT, Czech MP, Corvera S (2012) What causes the insulin resistance underlying obesity? Curr Opin Endocrinol Diabetes Obes 19: 81–87. 22327367 10.1097/MED.0b013e3283514e13 PMC4038351

[43] Premanath M, Basavanagowdappa H, Mahesh M, Suresh M (2014). Correlation of abdominal adiposity with components of metabolic syndrome, anthropometric parameters and Insulin resistance, in obese and non obese, diabetics and non diabetics: A cross sectional observational study. (Mysore Visceral Adiposity in Diabetes Study). Indian J Endo crinol Metab 18: 676–682. 10.4103/2230-8210.139231 PMC4171891 25285285

[44] Premanath M, Basavanagowdappa H, Mahesh M, Babu D MS, da Devanan (2016) Chronic sub-clinical inflammation in the abdominal adipose tissue-Evaluation of inflammatory cytokines and their link with insulin resistance in metabolically obese South Indians: A cross-sectional obser vational study. Indian J Endocrinol Metab 20: 84–91. 26904474 10.4103/2230-8210.172244 PMC4743390

[45] Mutimura E, Hoover DR, Shi Q, Dusingize JC, Sinayobye JD, (2015) Insulin resistance change and antiretroviral therapy exposure in HIV-infected and uninfected Rwandan women: a longitudinal analysis. PLoS One 10: e0123936. 10.1371/journal.pone.0123936 PMC4400132 25880634

[46] Yki Jarvinen HSK, Koivisto VA, Nikkila E (1989) Severity, duration, and mechanisms of insulin resistance during acute infections.J Clin Endocrinol Metab 69. 10.1210/jcem-69-2-317 2666428

[47] Menard A, Meddeb L, Tissot-Dupont H, Ravaux I, Dhiver C, (2017) Dolutegravir and weight gain: An unexpected bothering side effect? AIDS 31(10): 1499–1500. 28574967 10.1097/QAD.0000000000001495

[48] Venter WDF, Moorhouse M, Sokhela S, Fairlie L, Mashabane N, (2019) Dolutegravir plus Two Different Prodrugs of Tenofovir to Treat HIV. New England Journal of Medicine. 10.1056/NEJMoa1902824 31339677

[49] Stein Schalkwijk AC, Best Brookie, Stek Alice, Hawkins David, Wang Jiajia, (2016) Pharmacokinetics of efavirenz 600 mg QD during pregnancy and postpartum. CROI 22 – 25 February. Boston. Poster abstract 433.

[50] Calabrese Sarah K, Underhill Kristen (2015) How stigma surrounding the use of HIV preexposure prophylaxis undermines prevention and pleasure: A call to destigmatize truvada whores.American Journal Of Public Health 105: 1960–1964. 26270298 10.2105/AJPH.2015.302816 PMC4566537

